# The cerebral palsy transition clinic: administrative chore, clinical responsibility, or opportunity for audit and clinical research?

**DOI:** 10.1007/s11832-014-0569-0

**Published:** 2014-04-12

**Authors:** Fiona Burns, Robbie Stewart, Dinah Reddihough, Adam Scheinberg, Kathleen Ooi, H. Kerr Graham

**Affiliations:** 1 Orthopaedic Department, The Royal Children’s Hospital, 50 Flemington Road, Parkville, VIC 3052 Australia; 2 Murdoch Childrens Research Institute, The Royal Children’s Hospital, Parkville, VIC Australia; 3 The University of Melbourne, Parkville, VIC Australia; 4 Victorian Paediatric Rehabilitation Service, The Royal Children’s Hospital, Parkville, VIC Australia; 5 Young Adults Complex Disability Clinic, St Vincent’s Hospital, Fitzroy, VIC Australia

**Keywords:** Transition, Cerebral palsy, Audit, Clinical research

## Abstract

**Purpose:**

The majority of children with orthopaedic conditions in childhood survive to adult life, and there is a need for many of them to transition to adult services. This includes children with disorders such as club foot or developmental dislocation of the hip as well as those with complex syndromic conditions, bone dysplasias or neuromuscular disorders such as cerebral palsy and myelomeningocele. In many tertiary paediatric centres, transition has become a formal process in which clinicians document and communicate the status of patients who have been under their care to ensure a smooth transfer to adult services. The purpose of this report is to support the need for clear communication when children with cerebral palsy transition to adult services and to suggest that this transition represents a significant opportunity for audit and clinical research.

**Methods:**

Some of the factors to be considered in developing a minimum data sheet for the transfer or transition of children with cerebral palsy to adult services are described.

**Conclusion:**

Using the model of adolescents with cerebral palsy transitioning to adult services, orthopaedic surgeons can be encouraged to develop similar methodology and documentation for many other conditions for the purposes of communication, facilitation of transition, audit and clinical research.

**Electronic supplementary material:**

The online version of this article (doi:10.1007/s11832-014-0569-0) contains supplementary material, which is available to authorized users.

## Introduction

Cerebral palsy (CP) is the most common physical disability affecting children in developed countries, with a median prevalence of 2.4/1,000 live births and no indication of any decrease [1]. In many paediatric orthopaedic units, CP is the most common diagnosis after trauma. Children with CP are managed by a multidisciplinary team which may include specialists in paediatric neurology, paediatric rehabilitation, developmental paediatrics, gastroenterology, respiratory medicine, endocrinology, general surgery, neurosurgery and orthopaedic surgery. A wide range of allied healthcare providers are usually involved, including physiotherapists, occupational therapists and speech therapists. The clinical phenotype associated with CP varies from children with mild hemiplegia who may never require an orthopaedic intervention, to those with severe spastic quadriplegia who develop multiple musculoskeletal deformities and may require repeated intervention [2].

Transition has been defined as “the planned move of young people from paediatric health providers to adult providers/services” [3]. We recognise that the need for transition and the process itself will vary significantly within and between jurisdictions. For example, increasing numbers of children’s hospitals in North America provide care and surgical intervention for young adults with a primary presentation of hip dysplasia or residuals of hip dysplasia treated in early childhood. In such well-organised institutions, transition may be deferred until the need for arthroplasty. Some centres offering hip preservation surgery will also provide access to arthroplasty when it is needed. However, in other centres, such as our own, transition is essential because of the volume of workload as well as the recognition that the adult providers may offer a more appropriate environment for adult patients. In the state of Victoria (Australia), approximately 120 children are born each year with CP, and almost all of them receive services and follow-up at The Royal Children’s Hospital, the only tertiary care specialised paediatric provider in the state. Simple arithmetic dictates that our work would grind to a halt without a transition process. Transition is complex and should be a guided educational and therapeutic process—not simply an administrative event. In this article we will put forward the view that it is also an excellent opportunity for clinical audit and for clinical research.

Over 90 % of children with CP live beyond their 18th birthday [4], and after receiving services from paediatric institutions for approximately two decades, they will transition to adult services. Excess mortality in CP is mainly seen at Gross Motor Function Classification System (GMFCS) level V, but the converse of this means that the majority of children are surviving well into adult life and will need to access adult services when their care at a children’s hospital has been completed [4, 5]. There is currently no accepted age cut-off for transition to adult services, but the importance of transition is increasingly recognised from the perspective of the person with CP, their family, their family physician, the tertiary paediatric hospital and the adult providers who may take over the individual’s care [6–10]. Formal transition arrangements commenced at The Royal Children’s Hospital, Melbourne 15 years ago, and the senior author was asked to participate in this process by the developmental medicine paediatric team. Several adult provider teams had been identified, and it was considered essential to provide documentation with respect to the child’s orthopaedic management for these providers. Over the subsequent 15 years, a simple data collection sheet was developed and consistently modified in the light of experience to provide a “minimum data set” for the transition of adolescents with CP to adult care. However, given the investment in orthopaedic management of these children, both ambulant and non-ambulant, it became obvious that transition could also represent an opportunity for measuring important outcomes, which in turn could contribute to audit and clinical research. With suitable modifications, this approach for the transition of adolescents with CP could be used for other conditions to facilitate communication with adult providers.

Adolescents with CP face multiple challenges in the transition to adulthood that are associated with leaving school and subsequently finding employment and independent living. In addition, adolescents must face the issue of clinical transition, an area in which many problems have been reported [6], including the lack of communication between different healthcare providers. Parents sometimes report a sense of diminished support and increased burden of care [7]. Both paediatricians and general practitioners have expressed concern about the differences in healthcare services between paediatric and adult providers, the lack of knowledge about adult services and the difficulty in finding “the right service for the right adolescent”. Adult-oriented healthcare sectors and tertiary children’s hospitals have markedly different subcultures. For example, in our centre, a group of three surgeons with an interest in the musculoskeletal problems of children with CP provide three dedicated clinics for the needs of these children, which are staffed with allied healthcare professionals that include a clinical nurse coordinator, physiotherapists and gait laboratory staff. In addition, a combined clinic is conducted monthly run by an orthopaedic surgeon in conjunction with paediatric rehabilitation physicians. There is also a complex movement disorder meeting in which the management of young people with complex needs is discussed for the specific purpose of triaging tone management services, including botulinum toxin therapy, selective dorsal rhizotomy and intrathecal baclofen (ITB) [11]. In addition, there are robust hip surveillance programmes and a dedicated clinic for the evaluation and management of children with complex spinal deformities [12]. In the adult orthopaedic culture, surgeons are organised by narrow areas of super specialisation with the “generalist” rapidly becoming a creature of the past. If a young adult with CP has problems related to the spine, the hips, the knees, the feet and the upper limbs, it is possible that the person might need to see several orthopaedic surgeons [10], with the generalists being the family doctor and/or a specialist in adult rehabilitation medicine [9, 10]. The progressive subspecialisation of adult orthopaedic services has led us to re-double our efforts during childhood to complete and stabilise as many of the potential musculoskeletal problems as possible, particularly hip displacement and spinal deformity. The actual process of transition is now a subject of increasing evaluation and study [13].

## The minimum clinical data set

The first requirement for successful transition is accurate information. Of itself this is insufficient, but it is a good starting point. Over the years we have found many factual errors and contradictory statements during our reviews of hospital records at the time of transition. For those children with severe involvement, records are often voluminous, numerous sub-specialists are involved in the child’s care and it is uncommon for accurate summaries to be made at intervals in the child’s life. Summarising these extensive records is not easy and resolving factual inconsistencies can be challenging. We suggest that the transition document be discussed with the adolescent and family members and a copy be provided to them so that they can have a role in managing their own “data”.

The transition document [Electronic Supplementary Material (ESM) Appendix 1] makes no presuppositions about adult models of care nor about the management which teenagers with CP may receive when they leave our services. Rather the data sheet reflects what we believe to be appropriate multidisciplinary care, with a strong emphasis on the musculoskeletal aspects, from birth to skeletal maturity. In the adult setting, there may be increasing emphasis on education, vocational training, relationships and sexual functioning and employment issues. There are also the very important areas of pain management, degenerative arthritis and premature ageing. However, it is important to note that the origins of some of the pain issues and the degenerative joint disease issues may have their genesis in orthopaedic management of childhood, including hip displacement, hypertonia, contractures, torsional malalignments and spinal deformity. Providing patients specific information on the management of these issues up to the point of transition may aid adult providers in anticipating the needs of the patient in front of them. It will then be up to the adult providers to provide the best evidence-based care according to the patient’s needs and the resources available.

## Patient demographics

Accurate demographic information is a simple and useful starting point. This should be followed by a clear description of the evidence for, or the cause of, the CP, the type of CP and its classification. Caring for a child with disability puts additional stresses and strains on family relationships, and a summary of any changes in parental relationships, custodial issues and involvement of respite agencies can also be helpful. With respect to these sometimes sensitive issues, confidentiality must be considered, and this is a further reason for discussing the transition document with the adolescent and family members.

## Is the diagnosis of CP fully established?

Currently the majority of children with suspected CP under our care receive a detailed diagnostic work-up, including brain imaging to determine the presence of a brain lesion. Approximately 90 % of children with CP have a cerebral lesion identified on a magnetic resonance imaging (MRI) brain scan and, in conjunction with the history and clinical findings, the diagnosis can be well established [14]. However, 10 % of children with a clinical diagnosis of CP have a normal MRI scan, and the possibility of other diagnoses remains. Diagnostic certainty or uncertainty should be communicated in the transition document.

## Description and classification of CP: gross motor function

**Fig. 1:**
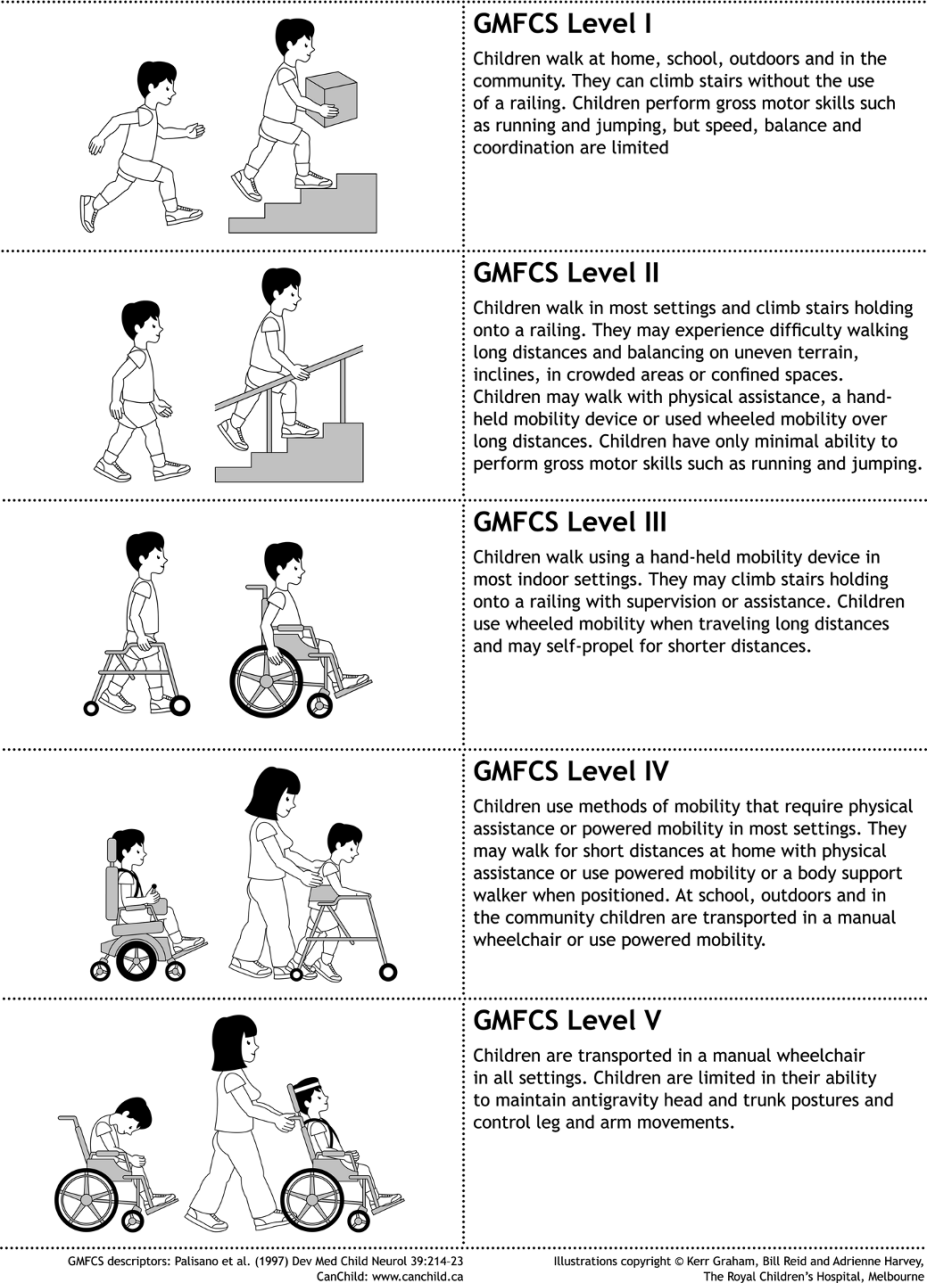
Expanded and revised Gross Motor Function Classification System (*GMFCS E & R*) for children from their 6–12th birthday: descriptors and illustrations

**Fig. 2:**
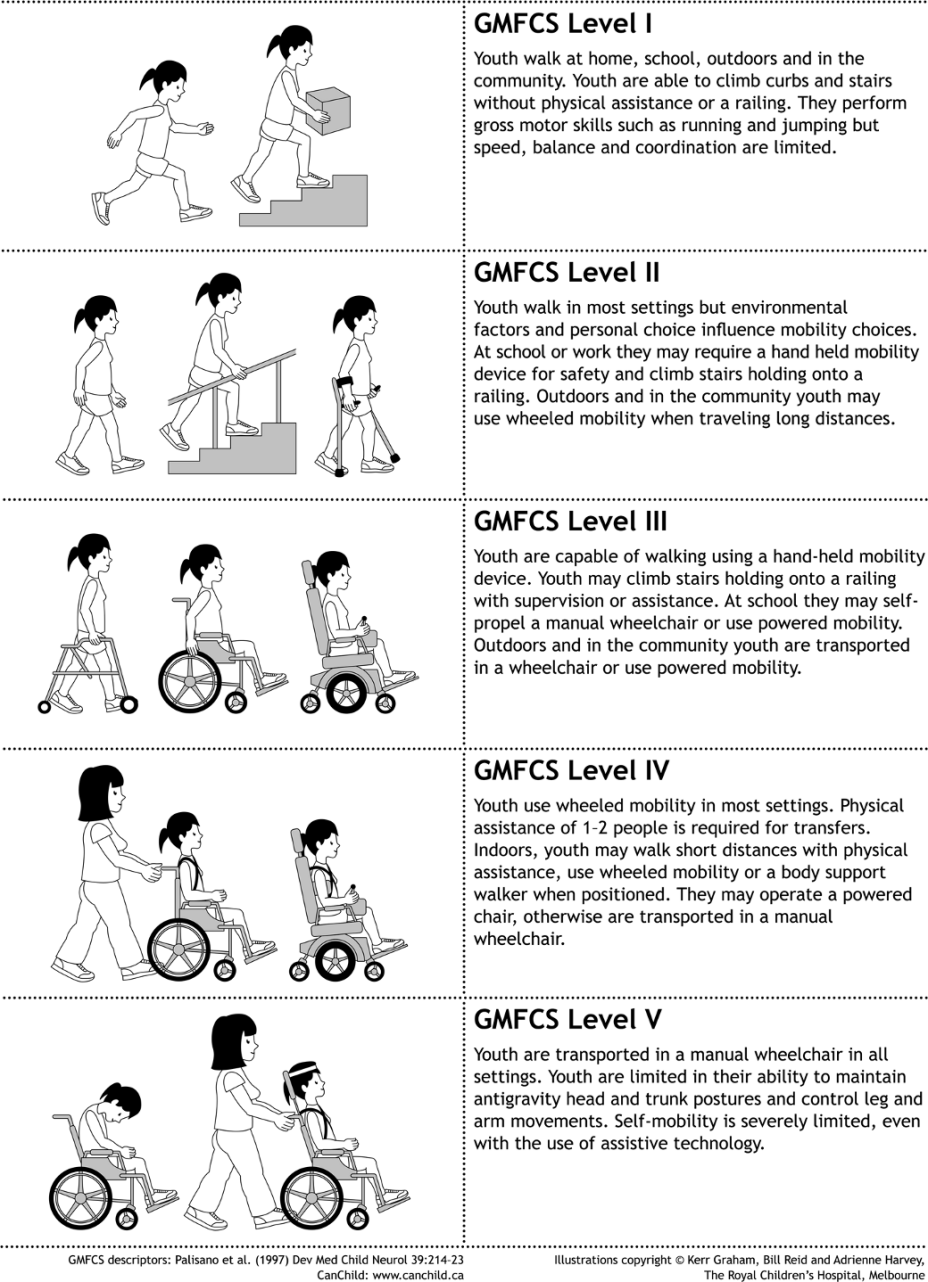
Expanded and revised Gross Motor Function Classification System (*GMFCS E & R*) for children from their 12–18th birthday: descriptors and illustrations

Classification of CP by the GMFCS has now become widespread although orthopaedic journals have been slower to adopt its use than developmental paediatric journals. It is difficult to communicate information on gross motor function in CP without using the GMFCS, and it is one of the most important aspects to report in a transition document [15, 16]. The GMFCS fulfils all of the psychometric properties required for a classification system, including validity, reliability, clinical utility and stability. In our service, the GMFCS level of approximately 90 % of children across all levels remains stable from first registration until transition [17]. GMFCS levels may change in response to improvements following interventions or deterioration consequent on interventions, or as part of the natural history of the disease. In our experience, the most common reason for a change in GMFCS status is an error in the previous or current GMFCS assignment [18]. Therefore, when a change in GMFCS level is noted, our first priority is to check if previous records have been correct. It is very important to establish the correct GMFCS level prior to transition and to document any changes and, if possible, the reason for such changes. It is important to note that there are significant differences in GMFCS descriptors and illustrations for children aged 6–12 years and for those aged 12–18 years. For the purposes of transition, most children are in the 12- to 18-year age group, and we therefore use these descriptors for the transition document (Figs. 1, 2).

More recently, newer classification systems have been introduced which in the next decade are likely to become as widely used as the GMFCS. These include the Manual Ability Classification System (MACS) and the Communication Function Classification System (CFCS) [19, 20]. These are both five level ordinal grading systems which have been consciously modelled on the GMFCS. The MACS is a grading system for bimanual upper limb function and is as relevant to upper limb function as the GMFCS is to gait function. The CFCS may be even more important. The management of such controversial problems as hip displacement and scoliosis depends to a significant degree on the patient’s awareness of these problems and the parental perception of their child’s distress. The individual’s ability to communicate pain, discomfort or distress associated with musculoskeletal deformities is likely to become a crucial part of assessment and guide management protocols in the future.

In terms of motor disorder, various approaches have been described, and there are important differences between those espoused in North America and Australia compared to Europe [21, 22]. Excellent descriptors of motor disorder classification systems have been published, but their reliability is not as good as those for gross motor function [23]. In addition, there is probably a great deal more true variability and change in motor disorder than in gross motor function. For example, many children who are classified as having spastic hypertonia in childhood are noted to have either dystonic hypertonia or mixed hypertonia when reassessed at transition. Hypotonia, which may be a feature of CP in early childhood, seems to evolve into various forms of hypertonia during childhood.

## Topographical classification

Orthopaedic surgeons continue to regard topographical classification as important because it focuses on the limb segments affected by the neurological deficits and by the hypertonia [2]. The preferred classification in Europe is unilateral and bilateral CP [22]; in North America and Australia, the terms hemiplegia, diplegia, triplegia and quadriplegia remain in use [2].

## CP function

In addition to classifying function using the GMFCS, gait function may be further classified using simple scoring systems, such as the Functional Mobility Scale (FMS) and the Gillette Functional Assessment Questionnaire [24, 25]. We have found these systems simple to use, reliable and sensitive to change following intervention and also with natural history. Similarly, with respect to the upper limb, in addition to the MACS, classifications of functional level according to the House Scale and the Shriner's Hospital for Children Upper Extremity Evaluation are also extremely useful [26, 27].

## Medical co-morbidities and history

In our transitional reports medical co-morbidities and history are frequently covered in much more detail by our colleagues in developmental medicine. Nonetheless, a fundamental understanding and summary by the orthopaedic surgeon of issues such as respiratory disease, nutritional issues and seizures are very important. Similarly, visual problems, hearing deficits, cognitive issues, learning disabilities, behavioural problems and mental health may impact on both the quality of life and responsiveness of the patient to orthopaedic interventions and should be therefore documented. The most common cause of mortality for adolescents at GMFCS IV and V is chronic lung disease, sometimes in conjunction with progression of scoliosis. Therefore, documentation of all admissions to hospital with episodes of pneumonia and to the intensive care unit for ventilation, as well as records of the need for home oxygen supplementation or various forms of ventilatory support, is extremely important.

## Tone management

The responsibility for tone management in tertiary centres varies; for example, injections of botulinum toxin may be administered in some centres by paediatric neurologists and in other centres by specialists in paediatric rehabilitation or orthopaedic surgeons [11]. Oral medications may be prescribed by a wide variety of healthcare professionals. Neurosurgeons perform selective dorsal rhizotomy, and ITB pumps may be inserted by orthopaedic surgeons or neurosurgeons. We believe it is important to record the utilisation and response to tone management, adverse events and future plans [28]. For example, some children are uniquely sensitive to injections of botulinum toxin and develop stridor and recurrent bouts of aspiration following the administration of doses which would be considered as standard and safe in other individuals [29]. The documentation of early response and later unresponsiveness to injections of botulinum toxin may indicate the development of neutralising antibodies or the progression of fixed muscle tendon contractures. Information from examination under anaesthesia as to the site, severity and significance of fixed muscle tendon contractures can be very useful to the adult rehabilitation physician in ongoing tone management in young adults. In our jurisdiction, future access to botulinum toxin under a government subsidised scheme is in part dependent on the documentation of its use in childhood. The need for clear documentation is therefore obvious.

Differences in both the availability and clinical practice of analgesia and sedation techniques in adult rehabilitation centres compared to paediatric rehabilitation centres may pose additional challenges for the continuation of Botox®.

## Surgical and anaesthetic history

Children with CP may have specific problems with anaesthesia, including difficulties with intravenous access and intubation, idiosyncratic responses to certain anaesthetic drugs and allergies to medications, dressings or skin preparation materials. Documentation of problems with previous anaesthetics is extremely helpful for a new management team in an adult tertiary care facility.

Information on feeding tubes, fundoplication and neurosurgical interventions, such as selective dorsal rhizotomy and ITB, are essential historical details. For those adolescents with an ITB pump in situ, details regarding the most recent programming of the ITB pump, the concentration of baclofen, planned refill date and catheter length are of crucial importance. It is important that all orthopaedic interventions and the dates of these interventions, as well as the response, rehabilitation and functional response of the patient, be documented prior to transition. For ambulant patients, records of three-dimensional gait analysis, gait patterns and changes relevant to tone management, the use of orthoses and orthopaedic surgery contribute to clear communication. In our centre, ambulant patients receive serial two-dimensional video recordings of gait from an early age and instrumented gait analysis (three-dimensional gait analysis) at intervals, including before selective dorsal rhizotomy, single-event multilevel surgery and at intervals thereafter [30]. When possible, we encourage a full three-dimensional gait analysis immediately prior to transition in order to advise the person with CP, their family and their future carers about functional status and expected future issues. In this “exit” or transition gait analysis, we describe fully the utilisation of assistive devices when appropriate, using the FMS [24]. In addition, we carefully compare gait parameters and function with and without orthoses, thereby providing adolescents and parents with the information—based on objective measurements—on just how much the current use of orthoses is helping gait and function. This approach provides additional information to the adolescent patient which he/she can base his/her personal choice for the ongoing utilisation of both assistive devices and orthoses. In this regard, serial examinations of composite gait indices including the Gait Profile Score are helpful [31]. At a glance, these scores all demonstrate whether gait function is static, improving or deteriorating [32].

## Radiology

**Fig. 3:**
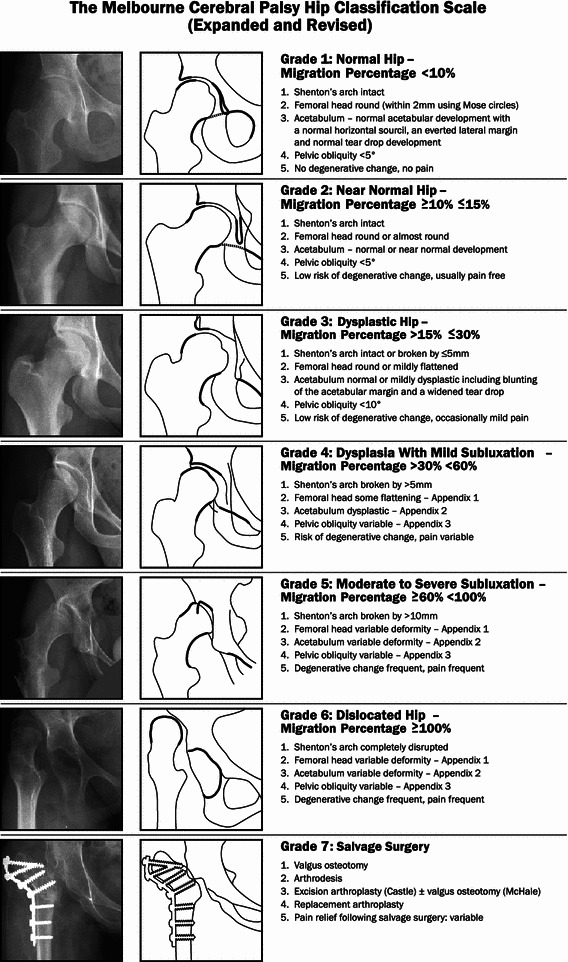
The Melbourne Cerebral Palsy Hip Classification Scale (MCPHCS) expanded and revised

**Fig. 4:**
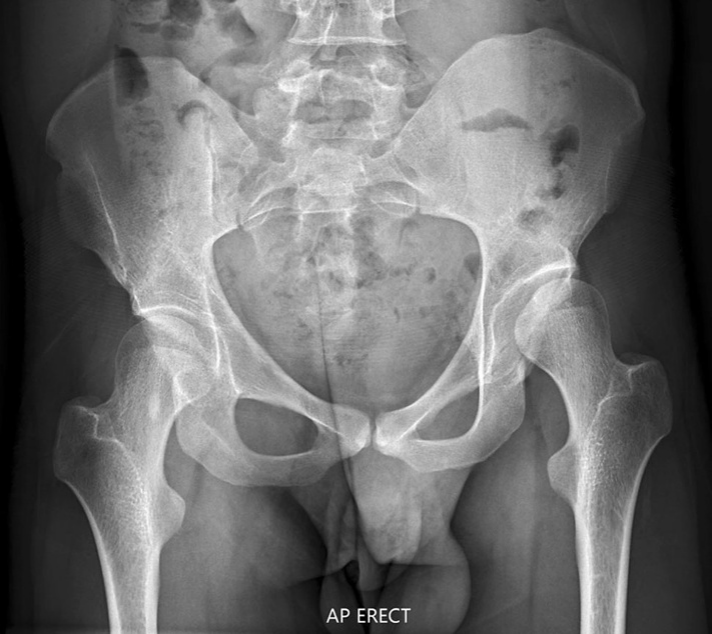
Anteroposterior (*AP*) pelvic radiograph of a young adult aged 21 years, GMFCS level III at the time of transition. Previous surgical history included bilateral adductor releases as part of single-event multilevel surgery to improve gait and functioning. At the time of transition there were no complaints of pain, and the patient walked well with a Functional Mobility Scale score of 5, 5, 5. The right hip is grade II according to the MCPHCS and will probably function well in adult life. However, the left hip is subluxated, dysplastic and is MCPHCS grade IV. Advice was given to continue with hip surveillance and to consider reconstructive surgery at the onset of any symptoms. Referral to an orthopaedic surgeon with an interest in hip dysplasia in young adults was also made

The most important imaging for patients prior to transition is plain radiology of the hips and spine. Hip displacement affects one-third of children with CP, and many children and adolescents receive surgical procedures to prevent or treat hip displacement [33, 34]. The trajectory of hip development in children and adolescents can be described using a variety of tools, including serial measurement of the migration percentage of Reimers, and at skeletal maturity can be classified using systems such as The Melbourne CP Hip Classification Scale (MCPHCS) [35]. It is important to communicate surgical complications, such as avascular necrosis and complications, that are relevant to the natural history of the disease, such as fixed deformity or the onset of early degenerative change, in a transition document. Just as important is the documentation of asymptomatic residual dysplasia or subluxation because these issues may become symptomatic in early adult life (Figs. 3, 4). For example, a significant number of young adults with type IV hemiplegia have mild, asymptomatic hip dysplasia on the affected side which is in part the result of pelvic obliquity [36]. Little is known about the impact of this subtype of CP hip dysplasia in the long term. However, given that individuals with hemiplegia are expected to have normal life expectancy, documentation of hip dysplasia and recommendations regarding follow-up are clearly important.

The prevalence of scoliosis is high in children and adolescents with CP, particularly those who are classified at non-ambulant GMFCS levels IV and V [37]. The presence, severity and prior treatment of scoliosis and other deformities, such as kyphosis and lordosis, require clear documentation. In addition, although scoliotic curves progress most rapidly during the pubertal growth spurt, spinal deformities may continue to progress in young adults with CP even after skeletal maturity [38]. Therefore, appropriate advice regarding long-term follow-up of spinal issues is important. Although some of the adolescents leaving our service have had a spinal fusion and are on a stable plateau following successful surgery, others have smaller curves and have not yet progressed to surgery. The provision of serial Cobb angle measurements to indicate both the magnitude and progression of spinal deformity can be very useful to those providing ongoing care. The presence of metal implants, such as blade plates in the hips or fixation rods in the spine, should also be communicated as this information may be relevant to future MRI of the spinal cord, especially if the instrumentation is stainless steel rather than titanium.

## Pain management

Recent studies have documented alarmingly high rates of pain in young adults with CP [39]. In some patients the pain is sometimes associated with specific issues, such as hip displacement, spinal deformity or foot deformity with early onset osteoarthritis [40], while in other patients, the pain is less well defined and may relate to the effects of chronic hypertonia, muscle exertion and fatigue [41]. At the time of transition it is important to document all complaints of pain, with precise descriptions of the severity, location and current management of the pain. The majority of adolescents with CP have good cognitive and communication abilities and can therefore usually provide a relatively straightforward description of the site and nature of the pain (ESM Appendix 2). In order to gain some simple information on the location, severity and frequency of pain, we use a generic Likert scale which is used across all GMFCS levels. The majority of young adults complete this scale quickly and easily during the transition appointment. At the other end of the spectrum, for adolescents with severe CP (GMFCS IV and V) and communication difficulties, who are at greater risk of underdiagnosis and undertreatment of pain, additional specialised tools are offered on a case-by-case basis. Fortunately, specialised tools are now available for the assessment and monitoring of pain in adolescents who are cognitively impaired [42].

## Quality of life and health-related quality of life

During transition, it is important to provide the adolescent patients and their parents or carers with the opportunity to express their feelings and give their own assessment of both their functional status and health-related quality of life. Some of the questionnaires which we have used in these patient groups include validated tools, such as the Child Health Questionnaire (CHQ), the Pediatric Outcomes Data Collection Instrument (PODCI), the Pediatric Evaluation of Disability Inventory (PEDI) and the CPCHILD© [43–46]. It can be very helpful to provide one of these questionnaires in advance of the Transition Clinic appointment. If the adolescent and/or parents bring the completed documentation to the appointment, it can be reviewed during the appointment, which facilitates the identification of areas of particular concern. For ambulant children with CP we use either the CHQ or the PODCI [43, 44]; for non-ambulant and more severely involved adolescents we use either the PEDI or the CPCHILD© [45, 46].

These questionnaires are also an excellent record at that point in time of various aspects of physical functioning and health-related quality of life which can then be used for the purposes of audit or for correlation with specific issues, such as hip or spine status.

## Bone health and fracture history

Impairment of bone health is very common in CP, and its importance is related to the high prevalence of fractures and bone deformity occurring in patients with CP. Apart from plain radiology, additional investigations which are increasingly widely used are dual-energy absorptiometry and peripheral quantitative computer tomography. The radiation exposure for both of these investigations is relatively modest, equating to a cross-country plane flight or about 2 % of the average background radiation per year. The fracture rate reported by Stevenson and colleagues in a longitudinal cohort study of 245 patients with moderate to severe CP was 15.7 % at baseline [47]. Twenty children reported 24 fractures during 604 person-years of follow-up, with 4 fractures/100 person-years (4 %/year). With a history of prior fracture at baseline, the fracture rate increased to 7 % per year. Having a gastrostomy tube (6.8 % per year) and higher body fat at baseline (9.7 % per year) were also associated with increased risk of fracture [47, 48]. Factors which may impact on a specific young adult’s bone health status—to be communicated in the transition document—include nutritional status, vitamin D and calcium levels, exposure (or lack of) to sunlight and the prescription of a number of anticonvulsants with anti-vitamin D effect, including Valproate. Details of current blood parameters and information on the prescription of supplements, including vitamin D, are important. Physical measures which may impact on bone health and fracture risk include weight-bearing status, the use of standing frames and low-frequency oscillation. Pharmacological management of bone health may include adequate nutrition, calcium and vitamin D supplementation and drugs such as bisphosphonates. Inclusion in the transition document of information on the history of bisphosphonate administration that documents both the effects and side effects of the treatment is important.

Given that a previous fracture is a major risk for further fracture, documentation of the timing, location and management of prior fractures is very important. Most fractures can be treated by simple means, including bulky dressings or well-padded splints. However, some fractures require complex fixation procedures, including intramedullary fixation. Long-term retention of intramedullary fixation rods may be helpful in the prevention of further fracture and secondary bony deformity [49].

## Research using transition documentation

When complete and accurate, transition documentation affords an excellent opportunity for audit and clinical research. This is the ideal time to document the long-term outcomes of tone management, gait correction surgery, preventive and reconstructive surgery for the hip and surgery for the correction of spinal deformities. The audit of hip status at skeletal maturity may be simply performed using the MCPHCS [35], and this simple tool may provide a year-by-year summary of the outcomes of hip surveillance, preventive surgery and reconstructive surgery. There are many additional areas in which the outcomes of specific interventions can be assessed in ethics-approved studies, including the outcomes of single-event multilevel surgery [32, 50]. Measures which may be of value in documenting the outcomes of single-event multilevel surgery, longitudinal changes and the effectiveness of the overall programme include the Gait Profile Score [31]. Orthopaedic surgeons should embrace the transition clinic as an excellent opportunity for clinical audit and potentially for clinical research at the crucial point in time when children and young people leave paediatric services.

## Electronic supplementary material

Below is the link to the electronic supplementary material.

Supplementary material 1 (DOCX 13 kb)

Supplementary material 2 (DOC 26 kb)
