# Supplementary material for: The cerebral palsy transition clinic: administrative chore, clinical responsibility, or opportunity for audit and clinical research?
Source: J Child Orthop. 2014 Apr 12;8(3):203–13. doi: 10.1007/s11832-014-0569-0 (PMC4142880; doi:10.1007/s11832-014-0569-0)
Supplement: Supplementary file 1 — Supplementary material 1 (DOCX 13 kb) [file 11832_2014_569_MOESM1_ESM.docx]

**APPENDIX 1**

**Date of Birth: Age: Date of Transition Assessment:**

**Diagnosis of cerebral palsy:** Clinical? MRI? Other Imaging?

**Classification of cerebral palsy:** GMFCS: I II III IV V, MACS: I II III IV V, CFCS: I II III IV V

**Topographical distribution:** Unilateral Bilateral

**Movement disorder:** Spastic hypertonia , mixed hypertonia, dystonia, ataxia, unclassified

**Gait pattern: Unilateral CP:** Winters, Gage and Hicks I, II, III, IV

**Gait pattern: Bilateral CP:** True equinus jump gait apparent equinus crouch gait

**Gait Function:** Functional Mobility Scale (FMS)

**Three Dimensional Gait Analysis:** Gait Profile Score (GPS): Gait Variable Scores (GVS):

**Tone management: Oral medications:** Type: Response: Side effects:

**Botulinum toxin (BoNT-A):** Target muscles: Dose: Effects: Side effects:

**Intrathecal Baclofen:** Date of pump implantation, current dose and programming details:

**Selective dorsal rhizotomy (SDR):** Date of surgery: Percentage of rootlet sectioned at each level:

**Paediatric surgery:** Insertion of feeding tube: Date: Type: Fundoplication: Date

**Nutritional status:** Current weight: BMI:

**Type:** Oral fed: PEG fed: Mixed:

**Respiratory Health:** History of hospitalisation: Admissions to ICU: Intubation: Requirement for supplemental oxygen.

**Bone Health:** History of fractures: vitamin D: administration: bisphosphonate administration:

**Gait Related Orthopaedic Surgery:** Single level surgery: Date: Type:

**Single Event Multilevel Surgery:** Surgical prescription: Pre and postoperative Gait Profile Score:

**Hip Surgery: Preventive surgery**: Date: Type:

**Reconstructive surgery:**  Date: Type:

**Salvage surgery:** Date: Type:

**Presence of retained metal implants:** Location: Type

**Surgery for spinal deformity:** Preoperative Cobb angle: Fusion levels: Instrumentation type: Postoperative Cobb angle: Surgical adverse events.
