# Supplementary material for: The cerebral palsy transition clinic: administrative chore, clinical responsibility, or opportunity for audit and clinical research?
Source: J Child Orthop. 2014 Apr 12;8(3):203–13. doi: 10.1007/s11832-014-0569-0 (PMC4142880; doi:10.1007/s11832-014-0569-0)
Supplement: Supplementary file 2 — Supplementary material 2 (DOC 26 kb) [file 11832_2014_569_MOESM2_ESM.doc]

**APPENDIX 2**

**PAIN SCALE FOR CHILDREN WITH CEREBRAL PALSY**

Question No 1: Are you currently experiencing any pain?

Question No 2: Are you able to indicate where the pain is located?

Question No 3: Which part of the body and which side?

Question No 4: If your son/daughter has difficulty in communication, can you please help complete the scales below?

For each question, we want you to circle the best number that shows how you think your child feels in respect of pain and if they have pain, the frequency of the pain.

**Question No 1: What is the current level of pain that you have?**

**Please circle the appropriate number**

**1 2 3 4 5 6 7 8 9**

**____________________________________________________________________**

No pain Mild Moderate Severe The most

at all pain pain pain severe pain

**Question No 2: If you currently have pain, how frequently does it affect you?**

**Please circle the appropriate number**

**1 2 3 4 5 6 7 8 9**

**_____________________________________________________________________**

Never Rarely Sometimes Frequently Very Always

frequently constant

(One or two days (One or two (Most days (Every day) (All day and

per month) days per each week) and all night)

week)
